# Avelumab demonstrates promise in advanced NSCLC

**DOI:** 10.18632/oncotarget.22418

**Published:** 2017-11-13

**Authors:** Ariel E. Marciscano, James L. Gulley

**Affiliations:** James L. Gulley: Genitourinary Malignancies Branch, Center for Cancer Research, National Cancer Institute, National Institutes of Health, Bethesda, MD, USA

**Keywords:** antibody-dependent cell-mediated cytotoxicity, anti-PD-L1, avelumab, immune checkpoint inhibitor, non-small cell lung cancer

The JAVELIN Solid Tumor clinical trials group recently reported the results of a multi-center open-label phase 1b study (NCT01772004) evaluating avelumab in patients with progressive or platinum-resistant metastatic or recurrent non-small cell lung cancer (NSCLC) [1]. Avelumab, a fully human IgG1 monoclonal antibody (mAb) that binds PD-L1, is currently approved by the U.S. Food and Drug Administration (FDA) for the treatment of metastatic Merkel cell carcinoma and advanced urothelial carcinoma refractory to platinum-based chemotherapy. Avelumab has shown an acceptable safety profile and encouraging clinical activity across a spectrum of metastatic solid tumors [2].

Among the five currently FDA-approved anti-PD-1/PD-L1 agents, avelumab is unique due to its unmodified fragment crystallizable (Fc) domain which may potentiate antibody-dependent cell-mediated cytotoxicity (ADCC) via interaction with CD16 (FcɣRIIIa) on natural killer (NK) cells [3]. The importance of ADCC to the efficacy of anti-PD-1/PD-L1 mAbs has not been evaluated clinically. Preclinical work has shown that ADCC may augment anti-tumor immunity, particularly among anti-PD-L1 mAbs. Further, avelumab has been demonstrated to induce ADCC across a wide variety of human tumor cell lines and enhance antigen-specific T-cell responses *in vitro* [3]. However, there is theoretical concern that ADCC could also lead to lysis of PD-L1-expressing immune cells. Indeed, IgG4 isotype mAbs (nivolumab, pembrolizumab) were selected due to low affinity for FcɣR, and IgG1 isotype mAbs (atezolizumab, durvalumab) have undergone Fc modification to specifically mitigate ADCC. To address this concern, Donahue and colleagues recently performed a comprehensive analysis of the peripheral immunome of patients treated with multiple cycles of avelumab (many enrolled on NCT01772004) observing minimal modulation of PD-L1+ immune subsets after treatment [4]. This study also demonstrated that avelumab mediated lysis of tumor cells but not peripheral blood mononuclear cells *in vitro.*

With a median follow up of 8.8 months in the NSCLC dose-expansion cohort, the JAVELIN investigators enrolled 184 patients with stage IIIB or IV NSCLC with disease progression after prior platinum-based doublet therapy for metastatic disease across 58 academic centers [1]. Based on the safety analyses and pharmacokinetic (PK) and pharmacodynamic profiling of PD-L1 receptor occupancy from the phase Ia dose-escalation trial, a dose schedule of infusional avelumab 10mg/kg every two weeks until progression or toxicity was selected for this study and other JAVELIN trials [2]. This study population was heterogeneous and heavily pre-treated and patient selection was agnostic of tumor histology or PD-L1 expression. Furthermore, unlike other studies of PD-1/PD-L1 inhibitors, this study allowed enrollment of sicker patients beyond the second line setting (33% of patients were in ≥3^rd^ line of therapy) and with other driver mutations (EGFR or KRAS mutation, ALK translocation). Multiple studies across multiple treatment modalities including immunotherapy have shown lower response rates with more advanced disease / later lines of therapy.

Objective response rate (ORR) was 12% (*n* = 22), and 1-year progression-free and overall survival rates were 18% and 36%, respectively. Ongoing follow-up has confirmed an additional 4 responses, raising the ORR to 14.1% (*n* = 26). Accounting for these factors, ORR was similar (12%-14.1% *vs*. 14%-20%) in this study compared with randomized prospective studies evaluating other PD-1 or PD-L1 inhibitors in the second-line advanced NSCLC setting [5]. Avelumab was generally well-tolerated and the high-grade adverse event rate was consistent with a recent large meta-analysis of patients treated with PD-1 or PD-L1 checkpoint inhibitors (13% *vs*. 11.4%) [6]. Ultimately, immune-related adverse events led to treatment discontinuation in only 2% of patients underscoring the favorable and manageable toxicity profile of avelumab.

To explore potential biomarkers of response to avelumab, clinical responses were stratified by PD-L1 expression. A proprietary assay for PD-L1 expression (anti-PD-L1 rabbit mAb clone 73-10) using a cutoff value of ≥1% on tumor cells showed a progression-free survival benefit for PD-L1 positive patients relative to PD-L1 negative counterparts (12.0 *vs*. 5.9 months, hazard ratio 0.27-0.75). These findings are exploratory and hypothesis-generating at present but add to a growing compendium of literature attempting to clarify the appropriate use of PD-L1 expression as a predictive biomarker for patient selection. A preliminary exposure-response analysis of this cohort of NSCLC patients explored the relationship between PD-L1 expression and avelumab exposure (trough concentration after first dose) [7]. Interestingly, in the subset of high-exposure patients, increased PD-L1 expression was associated with higher ORR (25.4% - 42.9%). It is plausible that increased induction of ADCC may underlie this association between exposure, PD-L1 expression and ORR. Further, population PK modeling suggests that more intensive dosing regimens (i.e. 10mg/kg weekly) could provide additional clinical benefit by augmenting avelumab exposure [7].

In consideration of this heterogenous, heavily pre-treated, PD-L1 non-selected advanced NSCLC patient population, avelumab demonstrated a toxicity profile and anti-tumor activity similar to other anti-PD-1 and PD-L1 mAbs [1]. This work provides rationale to further exploit the unique capacity of avelumab to induce ADCC as an additional mechanism of anti-tumor immunity. Indeed, efforts utilizing engineered NK cells (expressing high affinity CD16 allele and IL-2) to further optimize avelumab-mediated ADCC are ongoing and in various phases of preclinical and clinical testing [8].

**Figure 1:**
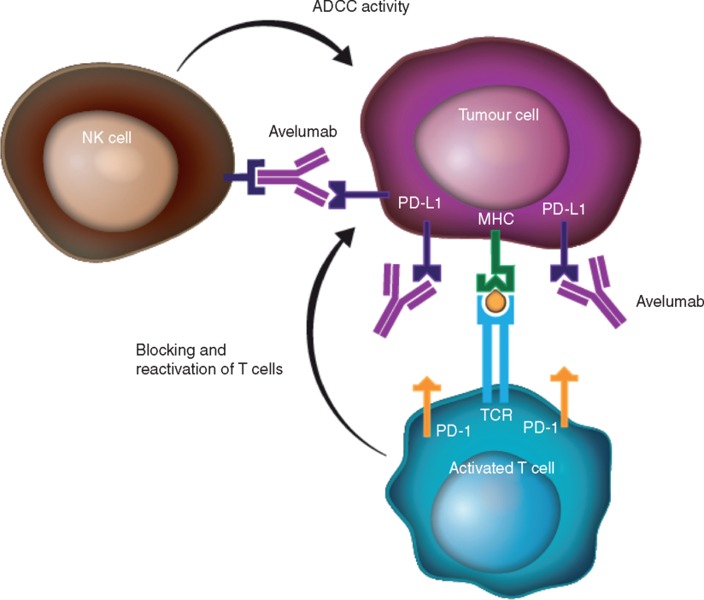
Dual mechanism of action of avelumab Avelumab, a fully human anti-PD-L1 IgG1 monoclonal antibody with an unmodified Fc region exerts anti-tumor immunity via: (1) blockade of the immune-inhibitory PD-1/PD-L1 interaction leading to reactivation of a T-cell mediated anti-tumor responses and (2) induction of natural killer cell-mediated antibody-dependent cellular cytotoxicity (ADCC) of tumor cells via interaction of CD16 (FcɣRIIIa) on NK cells with the Fc portion of the anti-PD-L1 mAb; Fc, fragment crystallizable; IgG1, immunoglobulin G1; MHC, major histocompatibility complex; NK, natural killer; PD-1, programmed death-1; PD-L1, programmed death ligand-1; TCR, T-cell receptor. Adapted with permission from Chin K, et al. Ann Oncol. 2017; 28: 1658-1666.
